# Ultrasound assessment of tibial nerve cross-sectional area in diabetic peripheral neuropathy in the Thai Population

**DOI:** 10.1371/journal.pone.0343128

**Published:** 2026-02-25

**Authors:** Athikhun Suwannakhan, Sittichai Khamsai, Thongchai Pratipanawatr, Woranan Kirisattayakul, Waranon Munkong, Nongnuch Prab Na Sak, Waritsara Phonsena, Sitthichai Iamsaard, Thanyaporn Senarai

**Affiliations:** 1 Department of Anatomy, Faculty of Science, Mahidol University, Bangkok, Thailand; 2 Human Anatomy Unit, Department of Biomedical Sciences, College of Medicine and Health, University of Birmingham, Birmingham, United Kingdom; 3 Department of Internal Medicine, Faculty of Medicine, Khon Kaen University, Khon Kaen, Thailand; 4 Department of Radiology, Faculty of Medicine, Khon Kaen University, Khon Kaen, Thailand; 5 Department of Rehabilitation, Faculty of Medicine, Khon Kaen University, Khon Kaen, Thailand; 6 Department of Anatomy, Faculty of Medicine, Khon Kaen University, Khon Kaen, Thailand; Debre Markos University, ETHIOPIA

## Abstract

Early recognition of DPN gives physicians the opportunity to deliver appropriate treatment and counseling to minimize subsequent complications. This study aimed to evaluate the diagnostic performance of tibial nerve cross-sectional area (CSA) in detecting DPN using a Modified Toronto Clinical Neuropathy Score (mTCNS) ≥ 3 as the diagnostic reference in Thai diabetic patients. A total of 67 diabetic patients (120 limbs) were enrolled from Srinagarind Hospital between 2022 and 2023. A total of 120 limbs belonging to 67 patients were categorized into two groups: non-DPN group (mTCNS < 3) (n = 42) and DPN group (mTCNS ≥ 3) (n = 78). Tibial nerve CSA was measured 3 cm proximal to the medial malleolus using ultrasound. Clinical parameters and metabolic profiles were recorded. Receiver operating characteristic analysis, correlation analyses, and multivariable logistic regression were performed to evaluate diagnostic utility and associations between CSA and clinical parameters. The tibial nerve CSA was significantly higher in the DPN group (13.49 mm^2^, 95% CI: 12.84–14.13) compared to the non-DPN group (11.98 mm^2^, 95% CI: 10.95–13.02) (p = 0.015). A CSA threshold of 13 mm^2^ yielded a sensitivity of 58.5% and specificity of 74.2%. CSA positively correlated with mTCNS (r = 0.49, p < 0.001) and sensation score (r = 0.37, p = 0.002) in DPN patients. Logistic regression identified CSA and estimated glomerular filtration rate as independent predictors of DPN status. Tibial nerve CSA may serve as a useful structural marker to support the identification of DPN. When used alongside established clinical assessments, CSA measurement could contribute to earlier detection and improved risk stratification in diabetic populations.

## Introduction

Diabetes mellitus (DM) is a chronic disease that poses a global public health challenge, with reports indicating that the incidence of DM is steadily increasing each year [1]. Similarly, in Thailand, the prevalence of DM rose from 6.9% in 2009 to 8.9% in 2014, and this trend is expected to continue [2,3]. It is well known that DM is often accompanied by complications if not properly treated, which significantly impact patients’ daily lives and economic conditions. These complications include retinopathy, chronic kidney disease, cardiovascular and cerebrovascular diseases, and complications in the hands and feet, known as diabetic peripheral neuropathy (DPN). Studies have shown that foot and leg complications are the most common complications among DM patients worldwide, affecting up to 20% of patients and leading to amputation of limbs in 40–50% of cases [4], causing disability and death. Early diagnosis before the onset of complications could enable physicians to provide timely treatment and guidance to prevent, reduce, or delay DPN-related complications [5].

Ultrasound is a diagnostic tool is recognized as a non-invasive, highly effective, low-cost procedure which can be used to detect nerve abnormalities and diseases [6,7]. Ultrasound offers a non-invasive and real-time assessment of nerve morphology, allowing visualization of structural changes such as nerve enlargement or altered echotexture in DPN. Unlike electromyography, ultrasonography can identify early anatomical alterations even before clinical symptoms or electrophysiological changes appear [6,7]. MRI neurography can also provide CSA measurements with high soft-tissue contrast but is less practical for routine use due to cost and time [8]. Previous studies [9–12] and a recent meta-analysis [13] has investigated the cross-sectional area (CSA) of the tibial nerve, demonstrating that DPN patients tend to have a larger CSA compared to diabetic patients without DPN. This enlargement of the tibial nerve CSA aligns with typical DPN symptoms, such as absent ankle reflexes and impaired sensations of vibration, pinprick, temperature, and light touch along the tibial nerve distribution. In contrast, peroneal and common peroneal nerve measurements are more useful in focal mononeuropathies and are less specific for the distal, length-dependent changes typical of DPN [14]. The distal tibial nerve demonstrates more pronounced CSA enlargement in length-dependent polyneuropathy than the sural nerve [15]. Despite the potential use of tibial nerve CSA as a diagnostic marker for DPN, a recent meta-analysis has recognized that tibial nerve CSA could be influenced by other factors such as ethnicity, age, weight, and body mass index, limiting its clinical utility [16]. Furthermore, no prior study has specifically examined the tibial nerve CSA in the Thai population.

Therefore, further research and population-specific data are needed to validate the feasibility of tibial nerve CSA as a potential diagnostic marker for DPN. The aim of this study is to investigate the CSA of the tibial nerve in DM patients with and without DPN in the Thai population.

## Methods

### Study population and neuropathy assessment

This study was approved by Khon Kaen University Ethics Committee for Human Research (HE651022). Participants were recruited from Srinagarind Hospital, Khon Kaen Province, between 19/09/2022 and 19/12/2022. Prior to participation, all volunteers provided written informed consent. Eligible participants were required to be over 18 years of age and capable of effective communication, excluding those who were unable to communicate due to advanced age or cognitive decline. Participants confirmed diagnosis of type 2 diabetes mellitus based on HbA1c results obtained within the past three months. The subjects could additionally exhibit clinical evidence of diabetic peripheral neuropathy, including a foot ulcer that did not interfere with tibial nerve ultrasound assessment. Participants were excluded if they had type 1 diabetes, a history of neuromuscular disorders or any other conditions affecting the peripheral nervous system. Additionally, limbs with ulcers or wounds on the soles of the feet or ankles, which could interfere with the study procedures, were not eligible for participation. Sample size was calculated using G*Power software based on a predetermined statistical power, effect size, and significance level. The suggested sample size was determined to be 125.

The sensory assessment in this study includes multiple tests to evaluate neuropathy status. The monofilament test (10g) was performed protective sensation by pressing a nylon filament against the foot. Failure to perceive it indicates sensory loss. The vibration test (128 Hz tuning fork) was used to measure vibratory sensation at the great toe and heel, with early loss suggesting neuropathy. The pinprick test evaluated pain sensation, while the cold sensation test assesses temperature perception using a cold rod. The proprioception test checked position sense by moving the great toe up or down with the patient’s eyes closed. The Modified Toronto Clinical Neuropathy Score (mTCNS) was used to quantify neuropathy severity by combining symptom assessment and sensory tests. Symptoms like pain, numbness, tingling, weakness, ataxia, and upper limb involvement are scored from 0 (none) to 3 (severe). The mTCNS also integrates monofilament, vibration, pinprick, cold sensation tests, and reflex assessments to classify neuropathy severity. In addition, fasting blood sugar (FBS), HbA1C, estimated glomerular filtration rate (eGFR), low-density lipoprotein (LDL), and triglycerides levels are recorded for all participants.

In total, 67 patients were enrolled, comprising 120 lower limb sides. Some limbs were excluded from the study because due to prior amputation or severe foot ulcers. Based on the mTCNS, participants were categorized into two groups: the non-DPN group (mTCNS < 3) (42 limbs) and the DPN group (mTCNS ≥ 3) (78 limbs). This classification was used as the gold standard for diabetic peripheral neuropathy diagnosis [17].

### Ultrasonography of the tibial nerve

**Fig 1:**
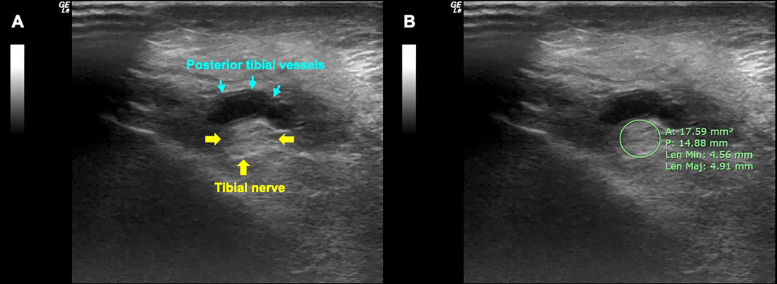
High-frequency ultrasound at the ankle showing the tibial nerve (A) and measurement of its cross-sectional area (B). Yellow arrowheads indicate the posterior tibial vessels and the red arrows indicate the boundaries of the tibial nerve.

Participants were asked to lie on their backs in a relaxed position. For the right tibial nerve measurement, the right leg was rotated to lateral position. The tibial nerve level located 3 cm proximal to the apex of the medial malleolus was used for measurement. This site was the most commonly used location for tibial nerve measurement, as indicated by our previous meta-analysis [13]. The ultrasound was performed by 2 well-trained radiological technologists, who were blinded to the DPN status. The ultrasound imaging was conducted using a linear array transducer (12L-RS, GE Healthcare, USA) in brightness mode. The transducer was placed transversely and perpendicular to skin. The ultrasound imaging parameters, including frequency (10 MHz), imaging depth (3.0 cm), gain (12 dB), focal zones (two points), dynamic range (75 dB), grayscale mapping (H/0), and frame rate (15 Hz), were set by an ultrasound product specialist to ensure optimal image quality (Fig 1). These parameters were kept constant across all participants. The left tibial nerve measurement was measured later following the same procedure. For each limb, two images were acquired by each observer, and the CSA values from the paired images were averaged to yield an observer-specific mean. The final CSA used for analysis was obtained by averaging the measurements from both observers to ensure consistency and reduce inter-observer variability.

### Statistical analysis

To explore associations between CSA and clinical parameters, Spearman’s rank correlation analysis were performed. Prior to the correlation analysis, Shapiro–Wilk test was performed to check data normality. A multivariable logistic regression analysis was performed to identify independent predictors of DPN among all study participants. The model was fitted using the statsmodels library in Python, and odds ratios (OR) with 95% confidence intervals (CI) were calculated by exponentiating the regression coefficients. Inter-rater reliability was assessed using the intraclass coefficient (ICC). Statistical significance was established at p = 0.05.

## Results

Demographic and clinical characteristics of participants were compared between diabetic patients with mTCNS < 3 (non-DPN group) and those with mTCNS ≥ 3 (DPN group) (Table 1). Duration of diabetes was significantly longer in the DPN group (128.4 ± 80.2 months) compared to the non-DPN group (93.1 ± 90.8 months, p = 0.038). Additionally, eGFR was significantly lower in the DPN group (76.7 ± 25.1) than in the non-DPN group (90.5 ± 17.5, p < 0.01). No other significant differences were observed between the groups.

**Table 1:** Demographic data of subjects (120 limbs from 76 subjects) included in the present study based on diabetic peripheral neuropathy status.

Variable	Non-DPN (mTCNS < 3) (42 limbs)^a^	DPN (mTCNS ≥ 3) (78 limbs)^a^	P-value
Age (years)	56.9 ± 10.7	61.0 ± 12.6	0.067
Female (%)	52.4%	60.5%	0.507
Weight (kg)	74.4 ± 17.8	69.6 ± 13.6	0.137
BMI	28.4 ± 5.0	27.7 ± 4.6	0.434
Duration (months)	93.1 ± 90.8	128.4 ± 80.2	0.038
FBS (mg/dL)	158.0 ± 59.5	148.2 ± 54.5	0.376
Hba1C	8.6 ± 2.4	8.0 ± 1.4	0.185
EGFR	90.5 ± 17.5	76.7 ± 25.1	<0.01
LDL	90.3 ± 32.6	86.5 ± 31.5	0.544

^a^ Some limbs were not included due to prior amputation or severe foot ulcers, accounting for the difference between the number of subjects and the total number of limbs

**Fig 2:**
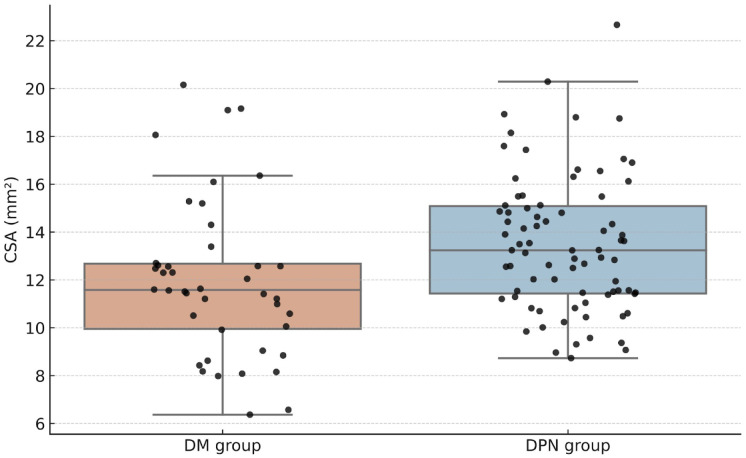
Comparison of tibial nerve cross-sectional area between diabetic patients without peripheral neuropathy (non-DPN group) and those with diabetic peripheral neuropathy (DPN group), classified according to the modified Toronto Clinical Neuropathy Score.

The mean tibial nerve CSA in the non-DPN group was 11.98 mm^2^ (95% CI: 10.95–13.02), while the DPN group had a significantly higher mean CSA of 13.49 mm^2^ (95% CI: 12.84–14.13) (p = 0.015) (Fig 2). The ICC was 0.81, indicating good agreement between the two observers.

**Fig 3:**
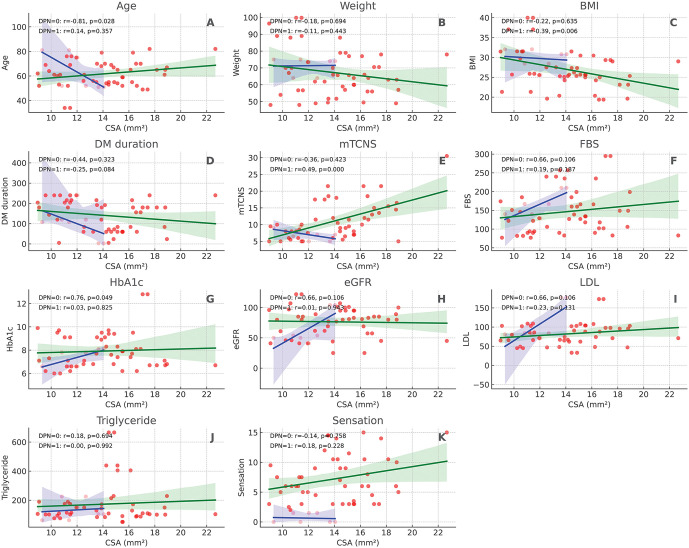
Correlation between tibial nerve cross-sectional area and clinical variables in diabetic (purple) and diabetic peripheral neuropathy patients (green). Each panel (A–K) displays a scatter plot with separate regression lines for both groups. Spearman correlation coefficients (r) and p-values are shown.

To evaluate the diagnostic performance of tibial nerve CSA in detecting DPN, CSA thresholds ranging from 7 mm^2^ to 19 mm^2^ were analyzed using mTCNS ≥ 3 as the diagnostic reference standard (Table 2). The optimal CSA threshold, determined by the highest Youden’s Index, was identified at 13 mm^2^. At this threshold, the sensitivity was 58.5% (95% CI: 46.5–69.7) and the specificity was 74.2% (95% CI: 64.3–82.3). Thresholds lower than 11 mm^2^ demonstrated higher sensitivity but considerably lower specificity, while thresholds above 15 mm^2^ markedly reduced sensitivity without substantial gains in specificity. Correlation analyses (Fig 3) between CSA and clinical parameters were performed separately for non-DPN and DPN groups. In the DPN group, significant positive correlations were observed between CSA and several neuropathy-related measures. CSA demonstrated a moderate positive correlation with mTCNS (r = 0.49, p < 0.001), indicating that nerve enlargement was associated with increasing neuropathy severity. Additionally, CSA was positively correlated with sensation score (r = 0.37, p = 0.002), suggesting that impaired sensory function corresponded with increased tibial nerve CSA. In the non-DPN group, CSA showed a significant positive correlation with fasting blood sugar (FBS) (r = 0.47, p = 0.004) and glycated hemoglobin (HbA1c) (r = 0.44, p = 0.007), reflecting a potential association between poor glycemic control and nerve structural changes even in patients without clinically overt neuropathy. No significant correlations between CSA and age, body weight, BMI, diabetes duration, eGFR, LDL, or triglyceride levels were identified in either group.

**Table 2:** Diagnostic performance of tibial nerve CSA thresholds detecting diabetic peripheral neuropathy (DPN).

CSA threshold (mm²)	Sensitivity (95% CI)	Specificity (95% CI)	Positive likelihood	Negative likelihood
7	100.0% (95.3–100.0)	4.8% (1.3–15.8)	1.05	0.00
8	100.0% (95.3–100.0)	7.1% (2.5–19.0)	1.08	0.00
9	97.4% (91.1–99.3)	21.4% (11.7–35.9)	1.24	0.12
10	91.0% (82.6–95.6)	26.2% (15.3–41.1)	1.23	0.34
11	80.8% (70.7–88.0)	35.7% (23.0–50.8)	1.26	0.54
12	65.4% (54.3–75.0)	54.8% (39.9–68.8)	1.45	0.63
13	52.6% (41.6–63.3)	76.2% (61.5–86.5)	2.21	0.62
14	39.7% (29.6–50.8)	78.6% (64.1–88.3)	1.85	0.77
15	25.6% (17.3–36.3)	81.0% (66.7–90.0)	1.35	0.92
16	19.2% (12.0–29.3)	85.7% (72.2–93.3)	1.35	0.94
17	11.5% (6.2–20.5)	90.5% (77.9–96.2)	1.21	0.98
18	7.7% (3.6–15.8)	90.5% (77.9–96.2)	0.81	1.02
19	2.6% (0.7–8.9)	92.9% (81.0–97.5)	0.36	1.05

A multivariable logistic regression model (Table 3) was developed to predict DPN status, defined by a Modified Toronto Clinical Neuropathy Score (mTCNS) ≥ 3. The predictors included tibial nerve cross-sectional area (CSA) and relevant clinical parameters, excluding mTCNS and sensation scores. In the full multivariable model, CSA (OR 2.01; 95% CI: 1.20–3.38; p = 0.008) and eGFR (OR 0.52; 95% CI: 0.31–0.87; p = 0.013) were significantly associated with DPN. Duration of diabetes, age, weight, BMI, FBS, HbA1c, LDL, and triglyceride levels were not significantly associated with DPN in the full model. Following stepwise backward elimination (Table 3), CSA (OR 1.95; 95% CI: 1.24–3.08; p = 0.004) and eGFR (OR 0.50; 95% CI: 0.32–0.80; p = 0.003) remained in the final model. Duration and other metabolic parameters were removed during the elimination process.

**Table 3:** A) Multivariable logistic regression predicting diabetic peripheral neuropathy, using cross-sectional area and clinical parameters with B) step-wise elimination.

Model	Variable	Coef.	SE	z	p-value	OR (95% CI)
Multivariable logistic regression	const	0.79	0.24	3.35	0.0008	2.21 (1.39–3.50)
	CSA	0.69	0.27	2.57	0.0096	2.01 (1.18–3.41)
	Non-DPN duration	0.57	0.27	2.08	0.0372	1.76 (1.03–3.00)
	Age	−0.38	0.34	−1.01	0.2719	0.68 (0.35–1.35)
	Weight	−0.79	0.45	−1.75	0.0802	0.46 (0.19–1.10)
	BMI	0.69	0.44	1.57	0.1161	1.99 (0.84–4.69)
	FBS	0.08	0.31	0.28	0.7774	1.09 (0.60–2.00)
	HbA1C	−0.32	0.32	−1.01	0.3098	0.73 (0.39–1.34)
	eGFR	−0.89	0.38	−2.34	0.0193	0.41 (0.19–0.86)
	LDL	0.24	0.24	0.99	0.3176	1.28 (0.79–2.07)
	Triglyceride	0.43	0.26	1.67	0.0952	1.55 (0.93–2.58)
Step-wise elimination	const	0.71	0.21	3.30	0.0010	2.04 (1.33–3.11)
	CSA	0.65	0.23	2.80	0.0051	1.92 (1.22–3.03)
	eGFR	−0.75	0.23	−3.18	0.0015	0.47 (0.30–0.75)

## Discussion

Our findings demonstrated that tibial nerve CSA was significantly greater in diabetic patients with DPN compared to those without, reinforcing its role as a potential structural biomarker for neuropathy. Our findings align with prior studies indicating that tibial nerve CSA is enlarged in DPN patients compared to diabetic controls without neuropathy [13,16,18–20]. Hypoechoic changes and increased nerve fascicle thickness on ultrasound have been well documented as early sonographic signs of neuropathy [19,21]. Standard diagnostic modalities for DPN, such as nerve conduction studies are valuable for assessing functional impairment but have well-recognized limitations. Nerve conduction studies are time-consuming, require specialized expertise, and often detect abnormalities only after significant nerve damage has occurred [12]. Quantitative sensory testing relies heavily on patient cooperation and cannot localize structural changes. Ultrasound measurement of tibial nerve CSA offers a rapid, non-invasive method to visualize structural alterations that may appear earlier in the disease course [6,7].

The mean CSA was significantly higher in the DPN group (13.49 mm^2^) compared to the non-DPN group (11.98 mm^2^) (p = 0.015). ROC curve analysis determined that a CSA threshold of 13 mm^2^ achieved the most favorable diagnostic balance, with a sensitivity of 58.5% and specificity of 74.2%. Threshold selection for tibial nerve CSA plays a crucial role in optimizing its clinical utility. According to Table 2, thresholds lower than 10 mm^2^ demonstrated higher sensitivity but substantially lower specificity, making them more suitable for screening purposes where the priority is to capture as many potential DPN cases as possible. Conversely, thresholds above 15 mm^2^ markedly reduced sensitivity without significant gains in specificity, indicating that they are better suited for confirmatory diagnosis where minimizing false positives is essential. In the context of previous ultrasound studies, the tibial nerve CSA values observed in our Thai cohort fall within the lower-middle range of those reported elsewhere. Our prior meta-analysis [13] demonstrated that mean tibial CSA in patients with DPN varied widely, from approximately 6–8 mm^2^ in early- or lower-stage disease [11,19,22,23] to more than 20 mm^2^ in cohorts with longer diabetes duration or more advanced neuropathy [10,18,24,25]. Intermediate mean values of around 12–19 mm^2^ were reported in several other series [26–31]. The pooled mean CSA for DPN across all studies was 15.1 mm^2^ (95% CI 11.8–18.5), with marked heterogeneity (I^2^ ≈ 100%). In comparison, the mean CSA of 13.49 mm^2^ in our DPN group and the optimal diagnostic threshold of 13 mm^2^ are slightly below this pooled estimate but remain broadly consistent with the lower end of the global range. While enlargement of tibial nerve CSA was associated with neuropathy, it does not capture all clinically affected individuals and may miss early or mild cases. In practice, this limitation suggests that tibial CSA should not be used as a standalone. It should be used alongside a complementary measure alongside established clinical assessments such as mTCNS or nerve conduction studies. Also, an elevated CSA may help identify patients who warrant closer monitoring or further electrophysiological evaluation, whereas a normal CSA does not exclude neuropathy. Factors such as age, BMI, metabolic status, and ethnic differences [13,16], which are known to influence peripheral nerve size, may further reduce the generalizability of a single cutoff value.

In terms of clinical correlations, CSA was significantly associated with mTCNS in the DPN group, reinforcing its role as a marker of neuropathy severity. However, no significant correlation was observed between CSA and the duration of diabetes in either group. This finding is in agreement with Oduola-Owoo et al. [32], who found no relationship between CSA and diabetes duration or BMI. Conversely, Jiang et al. [33] reported a positive correlation between diabetes duration and CSA especially in patients with disease duration exceeding 10 years. These differences may be attributed to variation in population characteristics, diabetes management practices, and the stage of neuropathy at the time of evaluation. With respect to glycemic control, our findings are consistent with some studies that found no significant correlation between HbA1c and tibial CSA or nerve mechanical properties [12,34]. Although previous studies suggested a positive association between HbA1c and nerve enlargement [18,24], our data indicate that CSA may reflect long-term structural damage rather than short- to mid-term glycemic fluctuations. Similarly, Hsieh et al. [35] demonstrated that elevated HbA1c levels were correlated with clinical and sonographic markers of polyneuropathy, but these associations did not translate into measurable increases in CSA. Interestingly, in our non-DPN group, CSA showed strong positive correlations with both HbA1c and eGFR. These findings suggest that subclinical nerve changes may be occurring in metabolically stressed individuals prior to the onset of clinical neuropathy. This is in line with emerging perspectives that view nerve enlargement as an early indicator of peripheral nerve stress, even before clinical thresholds for DPN are met.

While promising, this study has limitations. The relatively modest sample size may have limited the detection of smaller effect sizes in subgroup analyses. The cross-sectional design precludes determination of causality or temporal relationships between metabolic dysfunction and CSA changes. Potential confounders such as medication use, vitamin deficiencies, or comorbidities were not systematically controlled for. The operator-dependent nature of ultrasound measurements and the lack of electrophysiological correlation should be acknowledged. Because all participants were recruited from a single center, broader population inferences should be made with caution, and larger multicenter studies are needed to validate these findings. Future research is required to define tibial nerve CSA thresholds tailored to specific populations.

## Conclusion

Increased CSA was significantly associated with both neuropathy severity and reduced kidney function. A CSA threshold of approximately 13 mm^2^ provided the best diagnostic balance for detecting DPN, although its moderate sensitivity and specificity indicate that CSA should be interpreted as part of a multimodal assessment rather than a standalone diagnostic test. While tibial CSA shows promise as a structural marker that may aid severity classification, further longitudinal studies are required to determine its ability to predict neuropathy progression and to refine population-specific threshold values.

## References

[1] Saeedi P, Petersohn I, Salpea P, Malanda B, Karuranga S, Unwin N, et al. Global and regional diabetes prevalence estimates for 2019 and projections for 2030 and 2045: Results from the International Diabetes Federation Diabetes Atlas, 9th edition. Diabetes Res Clin Pract. 2019;157:107843. doi: 10.1016/j.diabres.2019.107843 31518657

[2] Chavasit V, Kriengsinyos W, Photi J, Tontisirin K. Trends of increases in potential risk factors and prevalence rates of diabetes mellitus in Thailand. Eur J Clin Nutr. 2017;71(7):839–43. doi: 10.1038/ejcn.2017.52 28422120

[3] Jaruratanasirikul S, Thammaratchuchai S, Sriplung H. Trends of childhood diabetes in Southern Thailand: 20-year experience in a tertiary medical center. World J Pediatr. 2017;13(6):566–70. doi: 10.1007/s12519-017-0049-y 29058250

[4] Vinik AI. Diabetic neuropathy: pathogenesis and therapy. Am J Med. 1999;107(2B):17S-26S. doi: 10.1016/s0002-9343(99)00009-1 10484041

[5] Thipsawat S. Early detection of diabetic nephropathy in patient with type 2 diabetes mellitus: A review of the literature. Diab Vasc Dis Res. 2021;18(6):14791641211058856. doi: 10.1177/14791641211058856 34791910 PMC8606936

[6] Kerasnoudis A, Tsivgoulis G. Nerve Ultrasound in Peripheral Neuropathies: A Review. J Neuroimaging. 2015;25(4):528–38. doi: 10.1111/jon.12261 25996962

[7] Padua L, Liotta G, Di Pasquale A, Granata G, Pazzaglia C, Caliandro P, et al. Contribution of ultrasound in the assessment of nerve diseases. Eur J Neurol. 2012;19(1):47–54. doi: 10.1111/j.1468-1331.2011.03421.x 21554493

[8] Vaeggemose M, Pham M, Ringgaard S, Tankisi H, Ejskjaer N, Heiland S, et al. Magnetic Resonance Neurography Visualizes Abnormalities in Sciatic and Tibial Nerves in Patients With Type 1 Diabetes and Neuropathy. Diabetes. 2017;66(7):1779–88. doi: 10.2337/db16-1049 28432188

[9] Kelle B, Evran M, Ballı T, Yavuz F. Diabetic peripheral neuropathy: Correlation between nerve cross-sectional area on ultrasound and clinical features. J Back Musculoskelet Rehabil. 2016;29(4):717–22. doi: 10.3233/BMR-160676 26966822

[10] Riazi S, Bril V, Perkins BA, Abbas S, Chan VWS, Ngo M, et al. Can ultrasound of the tibial nerve detect diabetic peripheral neuropathy? A cross-sectional study. Diabetes Care. 2012;35(12):2575–9. doi: 10.2337/dc12-0739 23033242 PMC3507587

[11] Tandon A, Khullar T, Maheshwari S, Bhatt S, Narang S. High resolution ultrasound in subclinical diabetic neuropathy: A potential screening tool. Ultrasound. 2021;29(3):150–61. doi: 10.1177/1742271X20958034 34567227 PMC8366217

[12] Watanabe T, Ito H, Sekine A, Katano Y, Nishimura T, Kato Y, et al. Sonographic evaluation of the peripheral nerve in diabetic patients: the relationship between nerve conduction studies, echo intensity, and cross-sectional area. J Ultrasound Med. 2010;29(5):697–708. doi: 10.7863/jum.2010.29.5.697 20427781

[13] Senarai T, Pratipanawatr T, Yurasakpong L, Kruepunga N, Limwachiranon J, Phanthong P, et al. Cross-Sectional Area of the Tibial Nerve in Diabetic Peripheral Neuropathy Patients: A Systematic Review and Meta-Analysis of Ultrasonography Studies. Medicina (Kaunas). 2022;58(12):1696. doi: 10.3390/medicina58121696 36556898 PMC9787041

[14] Narayan S, Goel A, Singh AK, Thacker AK, Singh N, Gutch M. High resolution ultrasonography of peripheral nerves in diabetic patients to evaluate nerve cross sectional area with clinical profile. Br J Radiol. 2021;94(1121):20200173. doi: 10.1259/bjr.20200173 33733810 PMC8506179

[15] Fisse AL, Katsanos AH, Gold R, Krogias C, Pitarokoili K. Cross-sectional area reference values for peripheral nerve ultrasound in adults: A systematic review and meta-analysis-Part II: Lower extremity nerves. Eur J Neurol. 2021;28:2313–8. 33794049 10.1111/ene.14850

[16] Senarai T, Suwannakhan A, Pratipanawatr T, Yammine K, Yurasakpong L, Sathapornsermsuk T, et al. Normative Reference Values of the Tibial Nerve in Healthy Individuals Using Ultrasonography: A Systematic Review and Meta-Analysis. J Clin Med. 2023;12(19):6186. doi: 10.3390/jcm12196186 37834829 PMC10573196

[17] Idiaquez JF, Alcantara M, Bril V. Optimal cut-off value of the modified Toronto Clinical Neuropathy Score in the diagnosis of polyneuropathy. Eur J Neurol. 2023;30(8):2481–7. doi: 10.1111/ene.15870 37203998

[18] Singh K, Gupta K, Kaur S. High resolution ultrasonography of the tibial nerve in diabetic peripheral neuropathy. J Ultrason. 2017;17(71):246–52. doi: 10.15557/JoU.2017.0036 29375899 PMC5769664

[19] Ishibashi F, Taniguchi M, Kojima R, Kawasaki A, Kosaka A, Uetake H. Elasticity of the tibial nerve assessed by sonoelastography was reduced before the development of neuropathy and further deterioration associated with the severity of neuropathy in patients with type 2 diabetes. J Diabetes Investig. 2016;7(3):404–12. doi: 10.1111/jdi.12408 27330728 PMC4847896

[20] Pop-Busui R, Boulton AJM, Feldman EL, Bril V, Freeman R, Malik RA, et al. Diabetic Neuropathy: A Position Statement by the American Diabetes Association. Diabetes Care. 2017;40(1):136–54. doi: 10.2337/dc16-2042 27999003 PMC6977405

[21] Singh KP, Kaur S, Arora V. Reference Values for the Cross Sectional Area of Normal Tibial Nerve on High-resolution Ultrasonography. J Ultrason. 2022;22(90):e144–52. doi: 10.15557/jou.2022.0024 36482929 PMC9714288

[22] Goyal K, Aggarwal P, Gupta M. Ultrasound evaluation of peripheral nerves of the lower limb in diabetic peripheral neuropathy. Eur J Radiol. 2021;145:110058. doi: 10.1016/j.ejrad.2021.110058 34839212

[23] Macaré van Maurik JFM, Schouten MEL, ten Katen I, van Hal M, Peters EJG, Kon M. Ultrasound findings after surgical decompression of the tarsal tunnel in patients with painful diabetic polyneuropathy: a prospective randomized study. Diabetes Care. 2014;37(3):767–72. doi: 10.2337/dc13-1787 24379356

[24] Wang F, Zheng M, Hu J, Fang C, Chen T, Wang M, et al. Value of shear wave elastography combined with the Toronto clinical scoring system in diagnosis of diabetic peripheral neuropathy. Medicine (Baltimore). 2021;100(35):e27104. doi: 10.1097/MD.0000000000027104 34477149 PMC8415960

[25] Zhong W, Zhang W, Yang M, Li G, Ma Q, Yang X. Impact of diabetes mellitus duration on effect of lower extremity nerve decompression in 1,526 diabetic peripheral neuropathy patients. Acta Neurochir (Wien). 2014;156(7):1329–33. doi: 10.1007/s00701-014-2087-8 24760499

[26] Breiner A, Qrimli M, Ebadi H, Alabdali M, Lovblom LE, Abraham A, et al. Peripheral nerve high-resolution ultrasound in diabetes. Muscle Nerve. 2017;55(2):171–8. doi: 10.1002/mus.25223 27312883

[27] Chen R, Wang X-L, Xue W-L, Sun J-W, Dong X-Y, Jiang Z-P, et al. Application value of conventional ultrasound and real-time shear wave elastography in patients with type 2 diabetic polyneuropathy. Eur J Radiol. 2020;126:108965. doi: 10.1016/j.ejrad.2020.108965 32268245

[28] Dikici AS, Ustabasioglu FE, Delil S, Nalbantoglu M, Korkmaz B, Bakan S, et al. Evaluation of the Tibial Nerve with Shear-Wave Elastography: A Potential Sonographic Method for the Diagnosis of Diabetic Peripheral Neuropathy. Radiology. 2017;282(2):494–501. doi: 10.1148/radiol.2016160135 27643671

[29] Dong Y, Li W, Yan G, Cheng J, Xian-jun M. The ROC curve analysis of the ultrasound characteristics of tibial neuropathy in patients with type 2 diabetes mellitus. J Hebei Med Univ. 2021;42:1032.

[30] He Y, Xiang X, Zhu B-H, Qiu L. Shear wave elastography evaluation of the median and tibial nerve in diabetic peripheral neuropathy. Quant Imaging Med Surg. 2019;9(2):273–82. doi: 10.21037/qims.2019.02.05 30976551 PMC6414758

[31] Kang S, Kim SH, Yang SN, Yoon JS. Sonographic features of peripheral nerves at multiple sites in patients with diabetic polyneuropathy. J Diabetes Complicat. 2016;30(3):518–23. doi: 10.1016/j.jdiacomp.2015.12.008 26782023

[32] Oduola-Owoo LT, Adeyomoye AA, Omidiji OA, Idowu BM, Oduola-Owoo BB, Odeniyi IA. Posterior Tibial Nerve Ultrasound Assessment of Peripheral Neuropathy in Adults with Type 2 Diabetes Mellitus. J Med Ultrasound. 2023;32(1):62–9. doi: 10.4103/jmu.jmu_13_23 38665340 PMC11040493

[33] Jiang W, Liao L, Lai Z, Li K, Luo W, Shen H. Ultrasound evaluation and grading of neuromuscular disease in lower extremities among diabetic patients. Am J Transl Res. 2024;16(7):3280–8. doi: 10.62347/WNSL1894 39114677 PMC11301481

[34] Pradhan DR, Saxena S, Kant R, Kumar M, Saran S. Shear wave elastography of tibial nerve in patients with diabetic peripheral neuropathy-A cross-sectional study. Skeletal Radiol. 2024;53(3):547–54. doi: 10.1007/s00256-023-04448-8 37698625

[35] Hsieh P-C, Ro L-S, Chu C-C, Liao M-F, Chang H-S, Kuo H-C. Relationship between nerve ultrasonography image and electrophysiology in diabetic polyneuropathy. J Diabetes Investig. 2025;16(2):257–64. doi: 10.1111/jdi.14353 39569559 PMC11786178

